# A new technique for tension-free reconstruction in large incisional hernia

**DOI:** 10.1007/s13304-017-0493-1

**Published:** 2017-10-13

**Authors:** Gabriele Munegato, Landino Fei, Michele Schiano di Visconte, Danilo Da Ros, Luana Moras, Gabriele Bellio

**Affiliations:** 1 Unit of General Surgery, Conegliano Hospital, Treviso, Italy; 2 Unit of Gastrointestinal Surgery, School of Medicine, Second University of Naples, Naples, Italy

**Keywords:** Abdominal wall, Large incisional hernia, Abdominal hernia, Mesh

## Abstract

In the surgical management of large incisional hernias, the main target is the closure of the abdominal wall defect on the midline without a dangerous increase in the intraabdominal pressure. In this setting, new intraperitoneal prosthesis and components separation techniques were proposed to solve this problem. Both solutions present some critical issues. A new surgical approach with a free lateral double layer prosthesis totally in polypropylene both sides (FLaPp^®^) is proposed to overcome this problem. This is a retrospective cohort analysis study with a prospectively collected database from two different Italian hospitals. Twenty-nine patients operated from April 2010 to December 2015 were treated using the new prosthesis. Four patients developed postoperative complications: one (3.4%) presented wound infection, two (6.9%) experienced seroma, and one had a hematoma (3.4%). No deaths were recorded. At a median follow-up of 28.5 months (IQR 22–36), no hernia relapse occurred. The application of FLaPp^®^ mesh is a safe and feasible option that can be employed to manage Rives repair in cases of abdominal wall defects with difficult closure of the posterior plan when the conventional prosthetic meshes could be unsuitable.

## Introduction

The reconstruction of complex and large incisional hernia (LIH), defined according to the definition of European Hernia Society [1], is challenging and technically demanding. Moreover, in the literature, there is no consensus regarding the best treatment, despite the new developments and the evolution of the existing surgical techniques [2]. In such instances, the main problem is how to solve the existing situation of “loss of tissue”. This term covers the combination of two factors: on one hand, the deficit of peritoneal and aponeurotic tissue; on the other hand, the diastasis of the abdominal wall muscle and the difficult reinsertion on the midline. Surgical management is affected by the pathogenic combination of these factors, which poses the issue of difficult closure of peritoneal and fascial edges without increasing the intraabdominal pressure, which would consequently reduce respiratory compliance and might cause severe postoperative respiratory complications [3, 4]. In addition, prosthetic fascial repair alone fails to obtain muscle reintegration on the midline with restoration of a satisfying abdominal wall physiologic dynamics.

This problem could be partially solved by creating a “neo-peritoneum” to induce a prosthetic enlargement of abdominal cavity without increasing the intraabdominal pressure. A possible solution could be achieved using a mesh as a “bridge” between the margins of the defect, but it has unacceptable rates of recurrence. Another way to solve this problem is represented by the IPOM (IntraPeritoneal Onlay Mesh) technique [5], which requires, however, the intraperitoneally implant of a large mesh, but this could lead to several possible complications. Nevertheless, both solutions fail to obtain a correct reinsertion of the rectus muscle on the midline.

A wide mobilization of the rectal muscle using the component separation technique associated with prosthetic repair appears to be the most effective and viable solution to solve both problems. This technique, first introduced by Ramirez in [6], and its later modifications [7, 8] currently finds great success, but these are complex procedures with wide areas of detachment and dissection. For what concerns anterior component separation, this invasiveness leads to high complication rate (20–43%) [9, 10] and relapse rate (15–22%) [10–12].

The development of a new surgical technique with a new composite prosthesis had made possible overcoming these crucial issues, simplifying considerably the prosthetic laparoplasty of LIH and allowing an adequate closure.

The aim of this study was to describe the experience with the new FLaPp^®^ mesh (Free Lateral Polypropylene Prosthesis–Dipromed srl, via Ciriè 22/a 10099 San Mauro Torinese, Torino, Italy), obtained by joining a layer of mesh in polypropylene monofilament and a layer of not-absorbable polypropylene film with anti-adherent properties, in the surgical management of LIH.

## Materials and methods

### Study population and selection criteria

We performed a retrospective cohort analysis with a prospectively collected electronic database in two hospitals in Italy (S. Maria dei Battuti Hospital—Conegliano, Treviso and Second University of Naples—School of Medicine—Naples). We included 29 adult patients operated with the FLaPp^®^ mesh from April 2010 to December 2015.

Preoperative preparation included informed consent, laboratory tests, and abdominal computed tomography (CT) scan.

Clinical characteristics including age, gender, body mass index (BMI), relevant comorbidities, and indication for repair were collected. The characteristics of the abdominal wall defects were recorded according to EHS classification [1].

Early postoperative complications were also collected. We defined early postoperative complication any adverse event occurring within the first 30 days after surgery. Follow-up clinical examinations were carried out at 1, 6, and 12 months after the operation. More clinical evaluations were performed on patients’ demand or according to the surgeon’s choice based on the patient’s risk of recurrence (e.g., obesity, wound infection…). Follow-up time was measured from the date of surgery to the last visit. The primary outcome was the presence of a recurrence during the follow-up. The recurrence was identified during follow-up visits or after referral to a consultant for signs or symptoms of recurrence. The diagnosis was made by clinical examination or abdominal CT scan if needed. The date of the diagnosis was recorded. The secondary outcomes were intraoperative and postoperative complications.

### Description of the new prosthesis

The FLaPp^®^ mesh is a new product in terms of material and morphology.

#### Material

It is a composite mesh made by polypropylene only as double layer. The innovation is made up in the different manufacturing process of polypropylene surfaces. A traditional upper layer, composed by macroporous monofilament polypropylene mesh to promote tissue growth and optimize colonization, is joined to a thin, smooth, lower layer of not-absorbable polypropylene film, which can be used in contact to the bowels [13]. In surgery is the first time that polypropylene is used in film fashion to minimize the adhesions. The ‘‘in vitro’’ study of polypropylene film showed that the new prosthesis, comprising the two layers, can be colonized by human fibroblasts on the side facing the abdominal wall, whereas the cells did not grow on the other side. The different morphology of the two layers aimed to favor cell growth on the upper side and to avoid adherence formation on visceral side [14, 15]. Moreover, as regards to the tensile strength of the polypropylene film, it has been demonstrated by mechanical tests that its resistance exceeds the thread’s one and the passage of the needle does not determine any tearing of the film itself. Vozzi et al. analyzed the influence of the topology of polypropylene mesh for abdominal wall repair, evaluating its ability to prevent and to minimize the formation of adhesions and to promote tissue ingrowths. Moreover, they found that the mechanical behavior of prosthesis presents an anisotropy index similar to that of natural tissue and a high safety index [16].

#### Morphology

**Fig. 1:**
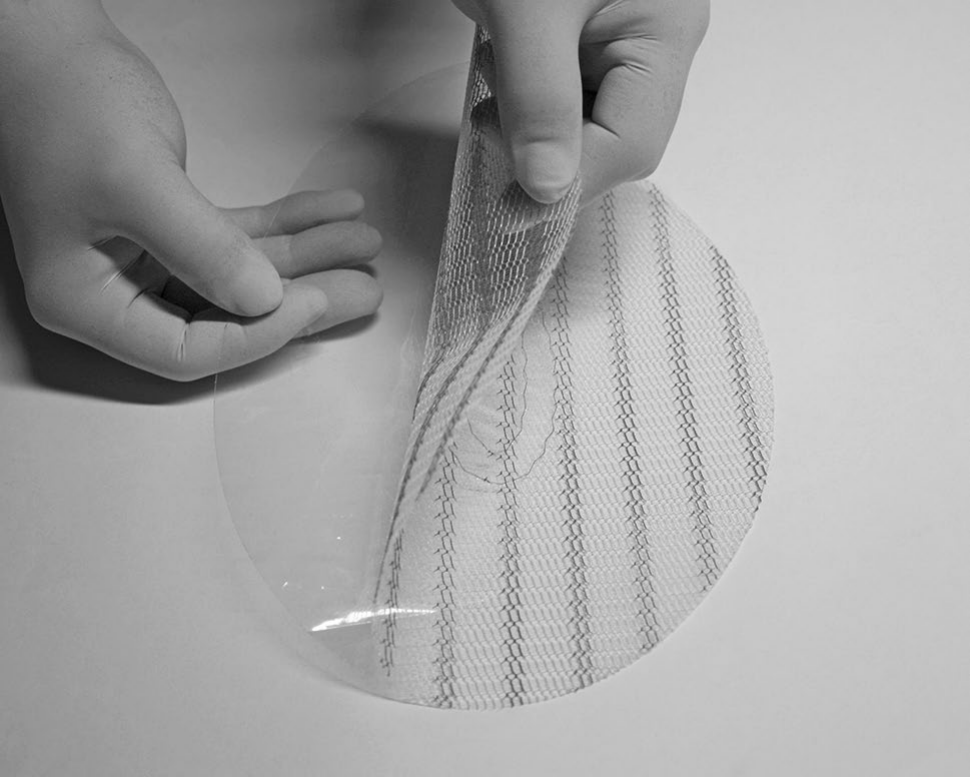
Composite FLaPp^®^ prosthesis with double prosthetic layer (lower in polypropylene film and upper in polypropylene mesh) joined in the central part creating two free flaps. The central part is oval and measures 4 × 7 cm. The peripheral portion of the prosthesis consists of two free flaps, the size of which is adapted to the defect

The prosthesis consists of two overlapping prostheses: upper layer, lightweight, macroporous polypropylene monofilament mesh (polypropylene mesh PM); and lower layer, transparent, non-porous polypropylene film (polypropylene film PF). These two layers are attached in a solid way in the central portion with a peripheral portion in which they can be easily separated from each other, creating two free flaps (Fig. 1). The central portion is oval and measures 4 × 7 cm, while the peripheral portion of the prosthesis consists of two free flaps, the size of which varies according to the size of the defect to repair.

### Surgical technique

Patients received antibiotic prophylaxis (Cefazolin 2 g, if not allergic) before the operation. All procedures were performed under general anesthesia and the patients were placed in supine position. The surgical technique was the same as described elsewhere [17, 18]. All patients had incisional hernia repair conducted by two surgical teams who used the same surgical approach. The same two expert surgeons [at least 30 complex abdominal wall repairs (cAWR) per year each] performed all the surgical procedures.

The surgical approach to LIH was the Rives technique with bilateral preparation of plans of dissection. The fascia of rectus muscle was incised along its medial border. Consequently, the dissection of the rear fascia of the rectus muscle was carried out up to the lateral margin of the muscle, where the vessels and ruptured nerves meet.

Above, near to the costal arch, the dissection returns to the peritoneal plan after a crosswise incision of the transverse and oblique-internal muscles. Behind, below the Douglas arch, the dissection returns to the peritoneal plan and it continues until it uncovers the symphysis pubis and Cooper’s ligament bilaterally [19, 20].

**Fig. 2:**
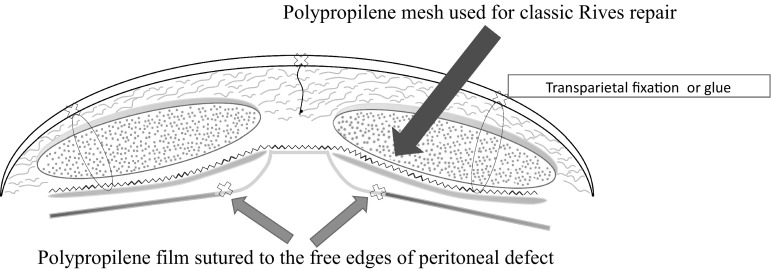
Schematic application of FLaPp^®^

The prosthesis was positioned according to the scheme shown in Fig. 2:

1. The PF is sutured to the margins of the residual peritoneum forming a “neo-peritoneum” in contact with the bowel loops. Short running resorbable suture is used adapting the PF to the shape and size of the defect (Fig. 3). Consequently, the PF in excess is excised. To avoid any misalignment of the mesh during the peritoneal suture, it is advisable to fix first the PM flap inferiorly to the Cooper’s ligament and superiorly below the xiphoid process.

**Fig. 3:**
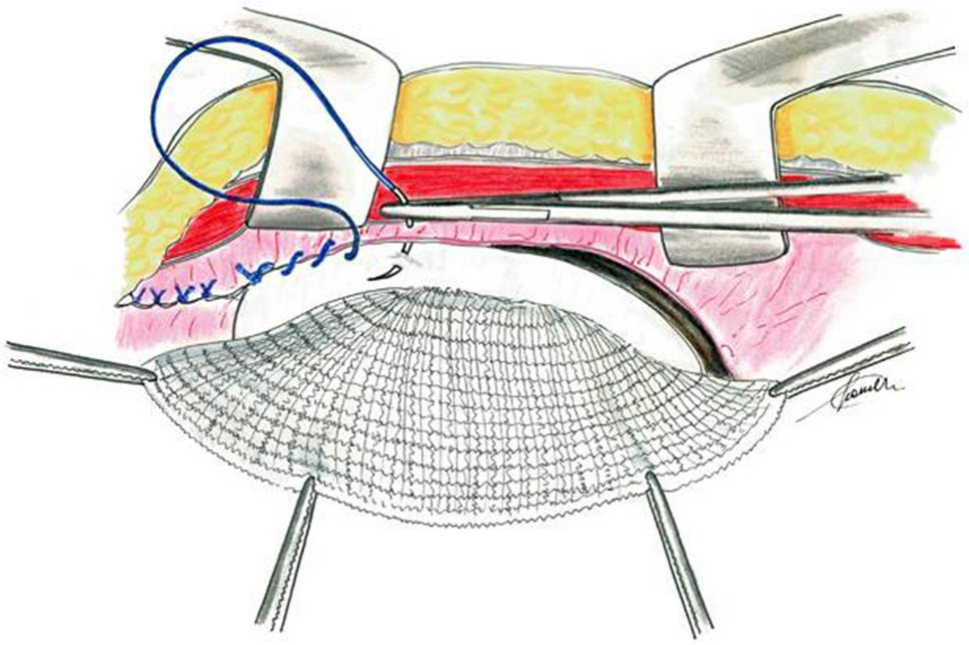
Polypropylene film flap is adapted to the shape and the size of the peritoneal defect and it is sutured to the free edges of the peritoneum with short running sutures. The polypropylene film can stay directly in contact with bowels
2. The PM is then used as a normal prosthesis mesh and it is placed in the previously prepared submuscular and prefascial area (Fig. 4). The PM needs to be shaped to suit the area of dissection between the rectus muscle and its rear fascia. The suture of the PF flap to the peritoneum and the two anchor points at the apical and caudal ends of the PM mesh already offers a stable framework for the prosthesis. Then, the prosthesis is fixed laterally with the traditional transfixed trans-parietal stitches through the full thickness of the abdominal wall. Alternatively, to avoid the postoperative pain due to transfixed stitches, it is possible to use the cyan acrylate glue.

**Fig. 4:**
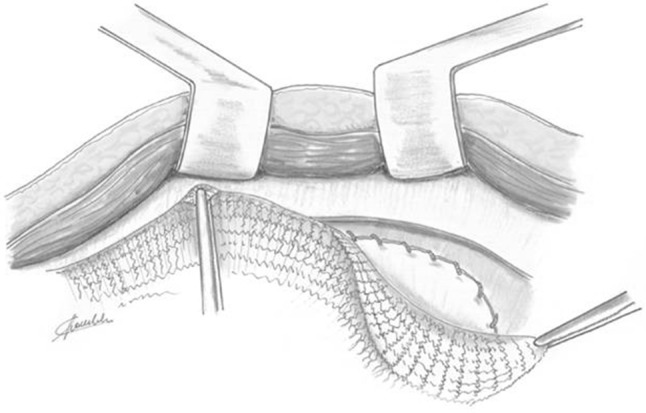
Polypropylene mesh is positioned in the area of dissection between the rectus muscles and its posterior fascia as a normal prosthesis mesh for Rives repair surgery
3. The residual superficial fascia is sutured with one running long-resorbable suture on the midline. This suture induces traction of the rectus muscles towards the midline and it separates the prosthesis from the subcutaneous tissue, reducing the risk of postoperative infection.

In all cases, the intraabdominal pressure was monitored during the surgical procedure by means of intravesical pressure measurements at basal time and at the time peritoneal closure.

### Statistical analysis

Data are expressed as median [interquartile range (IQR)] or number of patients (percentage).

## Results

The study population included 29 patients (18 women and 11 men) with a median age of 64 years (IQR 53–75); in particular, one-fourth was older than 75 years. Nineteen patients were operated at the Department of General Surgery in Conegliano; ten patients at the Unit of Gastrointestinal Surgery in Naples.

The preoperative clinical symptoms are shown in Table 1.

**Table 1:** Patients’ characteristics

Variables	Number of patients (%)
Gender	
Men	11 (38)
Female	18 (62)
Age (years)^a^	64 (53–75)
BMI	29.5 (27–34)
Number of previous hernia repair	7
Respiratory disease	4 (14)
Cardiovascular disease	10 (35)
Diabetes	5 (18)
Wound assessment	
Clean	24 (82)
Clean-contaminated	4 (16)
Contaminated	1 (2)
Dirty-infected	0

*BMI* body mass index

^a^Values are expressed as median (interquartile range)

The types of the abdominal wall defect according to EHS classification are shown in Table 2.

**Table 2:** Characteristics of incisional hernias

Variables	Number of patients (%)
Localization—Xipho-pubic (M1–M5)	29 (100)
Size–width	
4–10 cm (W2)	1 (3)
≥ 10 cm (W3)	28 (97)
Recurrent incisional hernia	7 (24)
Reducibility	
Reducible	9 (31)
Irreducible without obstruction	20 (69)
Previous repair technique	
Rives	6 (86)
Stoppa	1 (14)

Seven patients had the previous prosthetic laparoplasty. One patient had a W2 incisional hernia, but we decided to include him, as well, because the transverse diameter of the hernia was borderline (i.e., 9.5 cm). One male patient had a challenging picture of chronic obstructive pulmonary disease (COPD) and three female patients had a mild–moderate respiratory deficiency. Ten patients had cardiovascular disease. The median BMI was 29.5 (IQR 27–34). Moreover, five patients had a BMI higher than 39 and one of these patients had also an ischemic heart disease with aortic valve deficiency. The median mesh area was 170 cm^2^ (range 92–380) with median horizontal dimension of 10.2 cm (range 8–17 cm) and a median vertical dimension of 14.7 cm (range 8–26 cm).

The intravesical pressure measurements at the time of peritoneal closure did not recorded any increase in any of these patients.

There was no postoperative mortality. Table 3 summarizes the type of surgical morbidity. In particular, significant postoperative complications were observed only in one patient (3.4%) who experienced a hematoma requiring surgical intervention with partial detachment of the implanted device to control the bleeding and re-fixation of the same synthetic mesh. A Morales type 1 seroma [21] was observed in two patients (6.9%). One patient (3.4%) presented partial wound infection that resolved spontaneously. At a median follow-up of 28.5 months (IQR 22–36), no patient showed recurrence.

**Table 3:** Early and late postoperative complications

Complications	Number of patients (%)
Seroma	2 (6.9)
Hematoma	1 (3.4)
Wound infection	1 (3.4)
Recurrence	0 (0.0)

## Discussion

The surgical management of LIH is demanding, because the closure of the abdominal wall often poses several problems, such as difficulty in juxtaposing and directly suturing the peritoneal margins, interfacing the prosthesis deeply with the abdominal viscera or superficially with the subcutaneous tissue.

In the Rives technique, the site of incision of the fascia of rectus muscles, anteriorly or posteriorly, modifies the availability of peritoneal–fascial tissues with advantages and disadvantages. However, bigger hernia defects are, higher is the difficulty in approximating both the posterior and the anterior rectus sheath. In LIH, it is often impossible to close the prosthesis in a sure box between fascial plans, under and above.

In our experience, the incision of the fascia next to the medial margin of the rectum should be done on the rear side of the rectum, about 1 cm from its medial margin. This option has the advantage of avoiding placement of the mesh subcutaneously without any fascial covering, leading to high risk of infection. Petersen et al. [22] demonstrated the importance of closure of the rectus sheath ventral to the prosthesis during the retromuscular repair of incisional hernias. He found that the risk of deep prosthetic infection in patients in which it was not possible to close the rectus sheath above was 13 times higher than patients who received closure over the mesh. Carbonell et al. [7] reported that 2 patients out of 3 without anterior closure developed wound breakdown and mesh exposure compared to only one patient (5.8%) between those with anterior closure. Moreover, the preserved continuity and integrity of the anterior fascia of the rectum allows to approach it without difficulty along the midline with reintegration of rectum muscles on the midline.

On the other hand, this option does not resolve the problem of the posterior defect. In fact, a parietal closing “of necessity” entails an increase in intraabdominal pressure. The clinical relevance of this pathophysiological issue has been extensively demonstrated [23–25].

From a technical point of view, the setting of a valid prosthetic laparoplasty without increase of the intraabdominal pressure can be obtained by the IPOM technique [5], by creating a “neo-peritoneum” [23], by the preoperative progressive pneumoperitoneum (PPP) technique [26], or by component separation techniques [6, 8, 27, 28].

These methods have their drawbacks, which make them technically complex and expose them to criticisms.

In the IPOM technique, the amount of prosthetic material placed in the peritoneal cavity is considerable and the response in terms of visceral adhesions is unpredictable. Moreover, there is no tension on the fascial plan to allow the reinsertion of the abdominal wall muscles on the midline [5].

As for the use of absorbable or PTFE prosthesis as “neo-peritoneum”, it might be argued that it is a “bridge” repair, which has a significant percentage of structural failure to tensile forces. Moreover, there is a risk of development of seroma or hematoma in the space between the two meshes, and it is difficult to ensure a homogeneous tension on both the peritoneal and the fascial levels. Even with these solutions, the reinsertion of the rectum muscles on the midline remains unresolved [23].

A gradual increase in intraabdominal space by PPP acts as a useful adjunct in the treatment of LIH with “loss of domain”. The concerns regarding the use of PPP are subcutaneous emphysema, air embolism, and bacterial contamination [26].

The technique of components separation certainly enables a good reintegration of the rectus muscle on the midline with good functional results, but the procedure is complex and invasive with high rates of postoperative complications.

The large subcutaneous flap elevation is one of the major criticisms of the anterior component separation (ACS) described by Ramirez et al. [6]. This maneuver predisposes to large seroma and wound edges ischemia. In fact, the wound complication rate approaches 30% after Ramirez technique [27]. The recurrence rate up to 50% after the ACS imposed the use of a prosthetic repair (so that it would be correct to define as a “prosthetic” component separation). Even with mesh reinforcement, 1–5-year recurrence rate is about 10%. Recurrences and lateral bulging remain a problematic scenario and leave no easy surgical solution for these patients.

Posterior component separation (PCS) serves two purposes: medial mobilization of the transverse abdominis muscles with associated posterior rectus sheath and medial mobilization of the internal and external oblique muscles with accompanying anterior rectus sheath and muscles. This technique leads to less tension and to complete closure of the abdominal wall under the prosthetic mesh. This minimizes future bowel-to-prosthesis interactions and reduces the risk of adhesions, bowel obstructions, and enterocutaneous fistula formation. The main criticism to this technique is the access to the space between the transverse abdominis muscles and internal oblique muscles. This space contains the branches of the lateral cutaneous nerve, which, if damaged, would lead to lateral muscle paralysis and lateral-dorsal bulging [7].

Posterior component separation with transversus abdominis release (PCS/TAR) recently introduced by Rosen et al. [8, 28] showed advantages, in addition to those already mentioned for the PCS. This technique expects a dissection that preserves all the spraying and the innervation of the abdominal wall. The results are more than satisfactory with 28% of morbidity and 3.4% of recurrence rate. The difficulty and extension of the dissection, the difficulty to find a valuable peritoneal plan in pluri-operated cases, and the frequent presence of a very thin intact peritoneal plan could lead to reparation with double prosthesis (synthetic resorbable in combination with polypropylene).

However, the superiority of one technique over another has not been proved in large trials.

To solve these problems, we have suggested the use of a new mesh to treat LIH.

The surgical solution, achievable using the FLaPp^®^, appears a viable alternative to the component separation technique to obtain the closure of posterior peritoneal–fascial plan and to reinsert the rectus muscles on the midline. The use of the FLaPp^®^ is particularly indicated when the surgeon, after the preparation of the dissection plan according to the Rives technique, struggles with the closure of the lower fascial plan. Moreover, it is possible to use the FLaPp^®^ when there is a little posterior defect in which the PCS appears excessive, either for the surgeon’s lack of experience in this procedure or to not expose the patient to high and unnecessary risks of complications.

Clinical experience has confirmed the validity of the method which we used, with no recurrences or postoperative respiratory complications.

The advantages of this new prosthesis rely on its configuration. In fact, it enables a “tailored surgery” thanks to the two free flaps which can be shaped according to the hernia defect.

The PF is used as a neo-peritoneum, extending just enough to replace the shortage of the peritoneum, with a limited bowel loop-prosthesis contact and no peritoneal implant. It allows to enlarge “on demand” the abdominal cavity avoiding the risk of an abdominal compartment syndrome.

The PM, separated from the underlying PF, is placed on the plan of dissection between the posterior fascia and the rectus muscles, providing an optimal permeability for fibroblasts and connective tissue.

From a technical standpoint, the prosthesis is easy to fix using short resorbable suture connecting the PF and the margins of the peritoneal defect. The transparency of the mesh allows to control the underlying viscera providing a low risk of bowel injuries. Once the first surgical step has been completed, the prosthetic system is already stable and the use of cyanoacrylate glue alone is sufficient to fix it, without the need of transfixed stitches. This allows the prosthesis to adhere perfectly to the posterior fascia and to the rectus muscles, obliterating the dead space, and to avoid the risk of postoperative pain due to the entrapment of nerve-muscle fibers inside the stitches.

The fixation of the prosthetic system and the presence of a double mesh in its central portion confer tensile strength without any “swelling” due to parietal weakness. The function of the two meshes in the central portion reduces the risk of seroma which often characterizes implants with double separate meshes.

We believe that the FLaPp^®^ could find a compelling indication in patients with LIH and comorbidities, who require a surgical procedure as much as possible quick and safe, and in minor ventral hernias in patients whose clinical conditions (i.e., chronic bronchitis with respiratory deficiency, heart disease, and obesity) could increase the intraabdominal pressure.

The major limitations of our study are the small sample size, the short follow-up, and its retrospective nature. Further studies are needed to confirm our results and to compare this technique to others.

## Conclusions

The FLaPp^®^ prosthesis allows an easy and innovative approach for the treatment of LIH. The advantages can be summarized as follows:

- It reduces at minimum the interaction viscera–prosthesis and limits the formation of adhesions
- It is simple and easy learning
- It allows to avoid complex component separation techniques
- It reduces postoperative risks with advantages especially in patients with multiple comorbidities
- It may be used eventually along with the PCS/TAR, in case of pluri-operated patients or previous implant removal, where the closure of the defect on the midline appears to be difficult.
